# Integrative Single‐Cell Analysis Reveals Targetable Vacuole Membrane Protein 1‐Mediated Mechanism of Tumor Angiogenesis in Glioblastoma

**DOI:** 10.1002/mco2.70619

**Published:** 2026-01-25

**Authors:** Lei Jin, Bo Chen, Junbo Liao, Wenlong Guo, Zhiyuan Zhu, Salida Ali, Gilberto Ka‐Kit Leung, Peng Wang, Karrie M. Kiang

**Affiliations:** ^1^ Department of Neurosurgery Guangdong Provincial People's Hospital, Guangdong Academy of Medical Sciences, Southern Medical University Guangzhou China; ^2^ Department of Surgery School of Clinical Medicine LKS Faculty of Medicine The University of Hong Kong Hong Kong SAR China; ^3^ Department of Functional Neurosurgery Zhujiang Hospital, Southern Medical University Guangzhou China; ^4^ Queen Mary Hospital, Hospital Authority Hong Kong SAR China; ^5^ Clinical Neuroscience Consortium LKS Faculty of Medicine The University of Hong Kong Hong Kong SAR China

**Keywords:** angiogenesis, autophagy, glioblastoma, transmembrane protein, tumor microenvironment

## Abstract

Vacuole membrane Protein 1 (VMP1) is widely known to be an important mediator in the formation of autophagosomes, playing a crucial role in macroautophagic processes. Emerging evidence suggests that VMP1 may have context‐dependent functions across diverse cancer types and different tumor microenvironments, both within the context of autophagy and beyond. Here, using glioblastoma as a cancer model, we found that VMP1 can promote tumor growth independent of its autophagic functions. We observed significant upregulation of VMP1 in glioblastoma, which was correlated with poorer prognosis, and its ability to promote tumor growth without altering autophagic flux. Bulk, single‐cell, and spatial transcriptomics analyses revealed that the pro‐angiogenic markers were enriched in glioblastomas with high VMP1 expression. We further validated that overexpression of VMP1 would enhance angiogenesis through VEGFA‐VEGFR2 signaling‐mediated activation in endothelial cells. Treatment with bevacizumab, a monoclonal antibody against VEGFA, significantly inhibited VMP1‐driven tumor growth and prolonged survival in mice. Our study thus uncovered non‐autophagic functions of VMP1 as an important mediator in glioblastoma angiogenesis with the potential for therapeutic targeting.

## Graphical abstract

VMP1 promotes angiogenesis in glioblastoma (GBM) through VEGFA‐VEGFR2 signaling and activation of endothelial cells. The FDA‐approved anti‐VEGFA antibody, bevacizumab (BEV), can counteract VMP1's tumor‐promoting effect in GBM. Targeting VMP1 or its associated pathways could be a new therapeutic strategy, either alone or in combination with BEV. The diagram was created with BioRender.

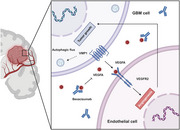

## 1 Introduction

Transmembrane proteins (TMEMs) are a family of proteins that transverse the lipid layer of plasma membrane or organelles, participating in numerous physiological processes and pathological conditions [1, 2]. Among these, vacuole membrane Protein 1 (VMP1), also known as transmembrane Protein 49 (TMEM49), is primarily located on the endoplasmic reticulum (ER) [3]. It was initially found to be a stress‐responsive protein in an acute pancreatitis model [4], and was later identified to be a crucial autophagic protein [5]. VMP1 has been reported to interact with Beclin 1 at contact sites between the ER and the plasma membrane, and it plays an important role in the formation of autophagosomes [6, 7]. Being a fundamental cellular process that involves the degradation and recycling of damaged cellular components [8], autophagy is critical for maintaining cellular homeostasis and for protecting against diseases [9, 10, 11]. However, cancer cells may exploit the process of autophagy to their survival advantages [12, 13], often through promoting metabolic adaptation and treatment resistance [14, 15].

Recent studies have demonstrated the dysregulation of VMP1 in various malignancies, but with conflicting results that appear to be organ‐specific and/or context‐dependent [16]. For instance, the upregulation of VMP1 has been implicated in cancer initiation as well as treatment resistance in pancreatic ductal adenocarcinoma through activation of autophagy [17, 18]. Aligning with this pro‐oncogenic role, VMP1 was found to be correlated with poor prognoses and overexpressed in both triple‐negative breast cancer and glioblastoma (GBM) [19]. By contrast, VMP1 expression levels have also been shown to negatively correlate with the invasive behavior of hepatocellular carcinoma [20, 21], suggesting an onco‐suppressive effect. Whether the tumor‐promoting effect of VMP1 is related to autophagy induction or is independent of autophagy is also not clear.

GBM is the most common and lethal primary brain tumor in adults. It is characterized by high basal autophagy, driven by its profound hypoxic microenvironment and the intrinsic autophagic dependency of glioma stem‐like cells [22]. Autophagy in GBM is constitutively active, serving as a major survival mechanism that supports cancer cell persistence and fuels resistance to the standard chemotherapy temozolomide [23]. Such reliance on autophagic pathways provides an ideal context in which to dissect VMP1 function, allowing for the elucidation of any autophagy‐independent activities when baseline autophagy is already maximally engaged. The present study aimed to interrogate the potential non‐autophagic functions of VMP1 in GBMs, based on the previous observation that while nearly all autophagic structures were co‐localized with VMP1 puncta, only around 5% of VMP1 puncta showed co‐localization with autophagic markers [24]. A considerable proportion of the remaining VMP1 puncta were closely associated with various organelles such as mitochondria, peroxisomes, endosomes, and lipid droplets in HeLa and COS‐7 cells [24], suggesting that VMP1 may be engaged in functions beyond autophagy.

Here, we report for the first time the pro‐angiogenic function of VMP1 in promoting tumor growth in GBM through VEGF‐A‐mediated endothelial cell activation. We demonstrate that VMP1 is overexpressed in gliomas and could confer survival advantages through enhanced angiogenesis, and that bevacizumab (BEV), a VEGFA‐targeted therapy, effectively suppresses VMP1‐driven tumor growth. These findings suggest that VMP1 could serve as a biomarker to identify GBM patients who are more likely to benefit from VEGFA‐targeted therapies, enabling more personalized treatment strategies. This study reveals a previously unrecognized interaction between VMP1 and the tumor microenvironment (TME) that is independent of its autophagic roles, highlighting both mechanistic and translational significance.

## 2 Results

### 2.1 VMP1 is Significantly Overexpressed in GBM and Associated With Poor Prognosis

**FIGURE 1:**
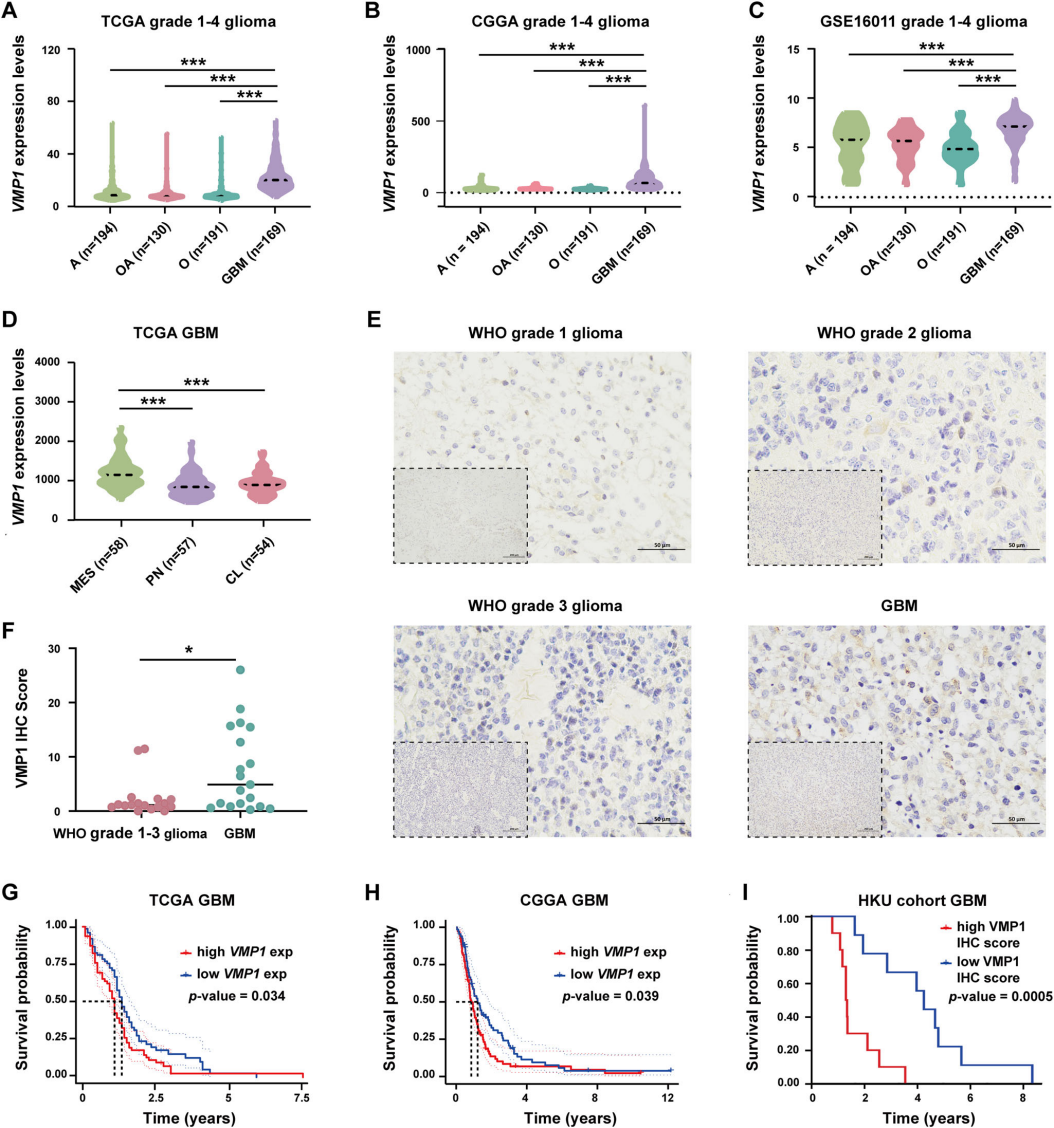
VMP1 is significantly overexpressed in glioblastoma with the association to poor prognosis. (A–C) VMP1 mRNA expression levels in different histological subtypes of glioma from TCGA (A), CGGA (B), and GSE16011 (C) datasets. Histological subtypes (A, astrocytoma; GBM, glioblastoma; O, oligodendrocytoma; OA, oligodendroastrocytoma). (D) VMP1 mRNA expression levels in different molecular subtypes of glioblastoma from TCGA datasets. Molecular subtypes (CL, classical; MES, mesenchymal; PN, proneural). (E, F) Representative immunohistochemistry (IHC) staining (E) and quantitation (F) of VMP1 expression in WHO 1–3 glioma (*n* = 17) and glioblastoma (*n* = 19) using in‐house IDH‐wildtype glioma specimens. Scale bar, 200 µm. (G) Kaplan–Meier survival analysis of glioblastoma patients with high (*n* = 81) and low (*n* = 81) VMP1 expression from TCGA datasets. (H) Kaplan–Meier survival analysis of glioblastoma patients with high (*n* = 70) and low (*n* = 69) VMP1 expression from CGGA datasets. (I) Kaplan–Meier survival analysis of glioblastoma patients with high and low VMP1 expression from in‐house cohort datasets. *p < 0.05; ***p < 0.001.

To gain insights into the clinical significance of VMP1 in human glioma, we examined the expression profiles of VMP1 in gliomas of different malignancy grades, histological subtypes, and molecular subtypes using data retrieved from TCGA, CGGA, and GEO. Gene expression analyses in tumor tissues (inclusive of both IDH‐wildtype and IDH‐mutant gliomas) revealed that VMP1 was significantly overexpressed in GBM (Figure 1A–C), the most malignant histological subtype [25]. VMP1 was highly expressed in mesenchymal GBMs (Figure 1D), a molecular subtype correlated with the worst clinical outcome [26]. To determine whether this was independent of IDH mutation, we examined our in‐house IDH‐wildtype glioma specimens and found a relative overexpression of VMP1 in GBM (WHO Grade 1–3 vs. WHO Grade 4 GBM, *p *= 0.013) (Figure 1E,F). With respect to the prognostic significance of VMP1, GBM patients with high VMP1 expression were found to have a shorter overall survival duration in all three cohorts (TCGA, *p = *0.034; CGGA, *p = *0.039; our in‐house cohort, *p *= 0.0005) (Figure 1G–I and Figure S1).

### 2.2 VMP1 Promotes Tumor Growth Independent of Autophagy

**FIGURE 2:**
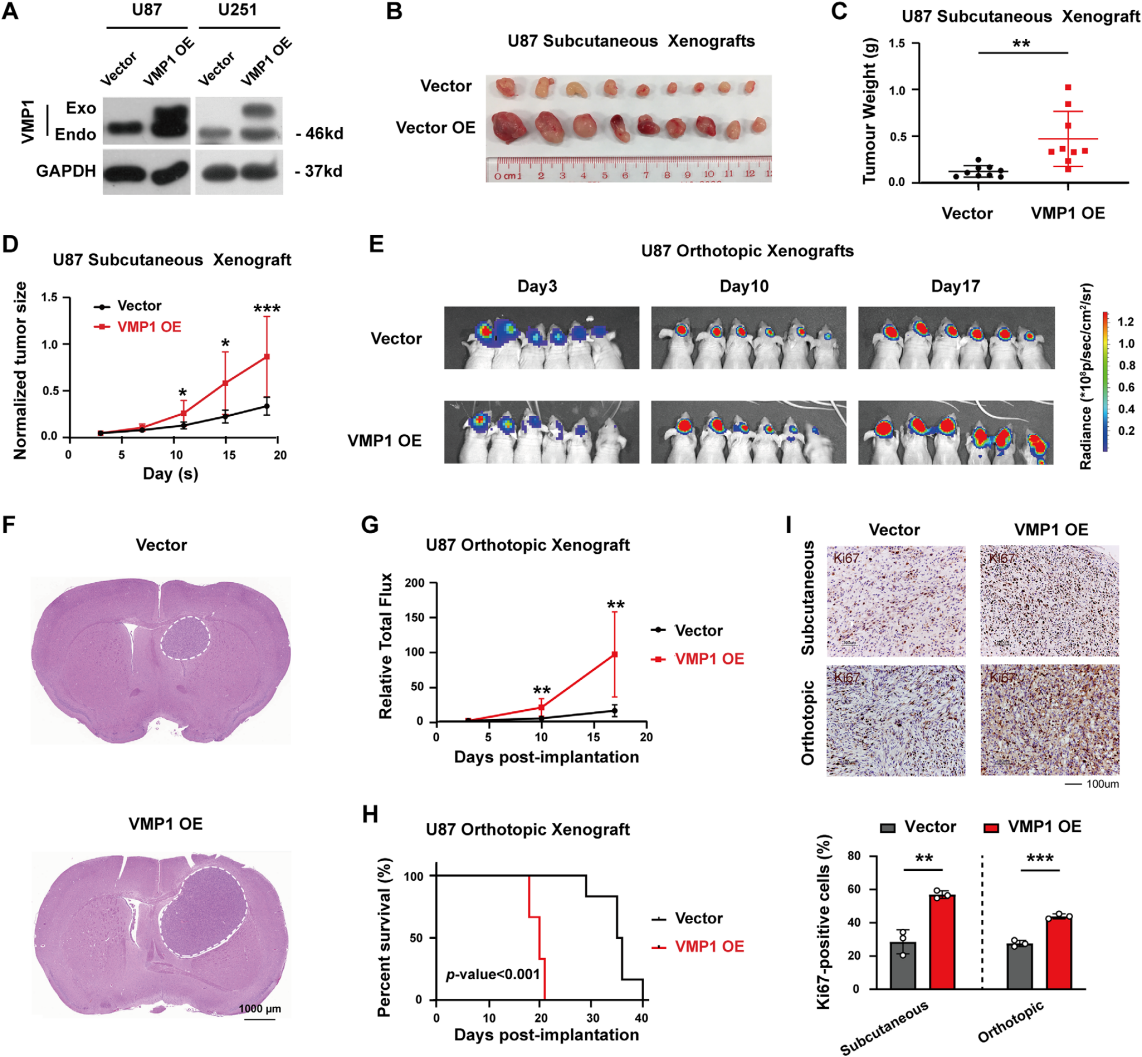
Overexpression of VMP1 promotes tumor growth. (A) Representative western blot image validating exogenous overexpression of VMP1 in U87 and U251 cell lines. (B) U87 subcutaneous xenografts of overexpressed VMP1 (VMP1‐OE) and control vector at Day 17 post‐injection (*n* = 9). (C) The weight of the tumor grafts. (D) Quantification of normalized tumor weights of VMP1‐OE and vector (measured every 4 days from Day 3) (*n* = 9). (E) Bioluminescence imaging of U87 VMP1‐OE and vector orthotopic xenografts at different time points from Day 3 to Day 17 (*n* = 6). (F) Representative images of hematoxylin and eosin‐stained sections at Day 17 post‐injection. Tumor is indicated within the dashed line. Scale bar, 1000 µm. (G) Relative total photon flux of bioluminescence in mice with U87 VMP1‐OE and vector. (H) Kaplan–Meier survival analysis of mice with U87 VMP1‐OE and vector intracranial xenografts (*n* = 6). (I) Top, immunohistochemical staining showing Ki67‐positive cells in subcutaneous and intracranial xenografts. Scale bar, 100 µm. Bottom, quantification of Ki67‐positive cells (%) in subcutaneous and intracranial models. *p < 0.05; **p < 0.01; ***p < 0.001.

To assess the functional role of VMP1 in GBM, we generated VMP1‐overexpressing U87 and U251 cells using the lentiviral system. Overexpression of VMP1 was confirmed with the expression of exogenous VMP1 protein expression (Figure 2A). We then used a xenograft model to determine the functional dependency of VMP1 in mediating tumor growth within different TME and found that GBM cells with VMP1 overexpression (VMP1‐OE) would promote subcutaneous tumor growth in mice (Figure 2B,D). Consistently, VMP1‐OE intracranial xenografts grew at a much higher rate than the vector group at 17 days post‐implantation (Figure 2E–G). Mice in the U87 VMP1‐OE group displayed a significantly shorter median survival time compared to those in the vector group (15 days for VMP1‐OE vs. 30 days for empty vector group, log‐rank *p *< 0.001) (Figure 2H). The tumor‐promoting function of VMP1 was also supported by the significant increase in Ki‐67 proliferation index in VMP1‐overexpressing tumors (Figure 2I).

**FIGURE 3:**
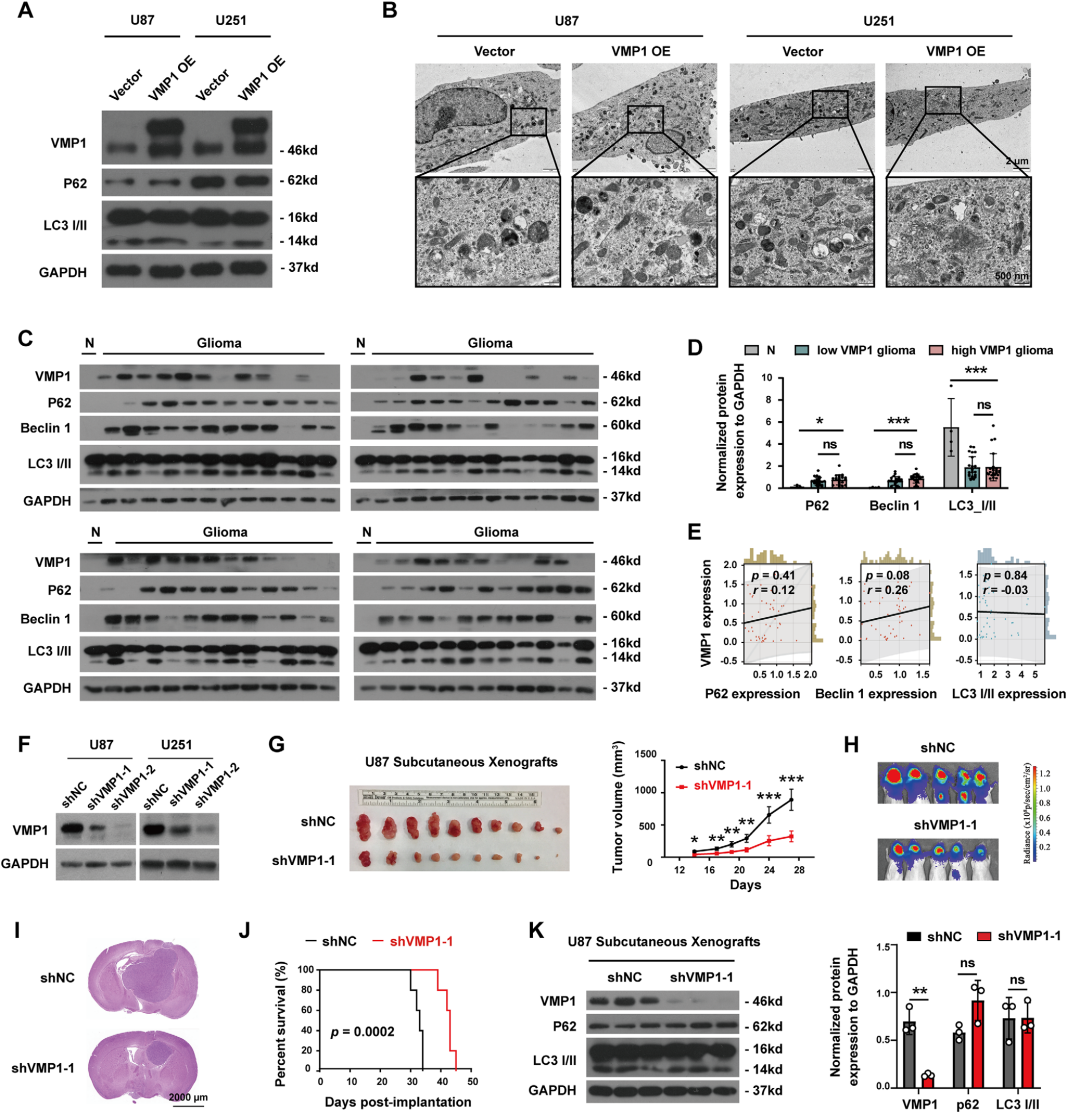
VMP1 promoted tumor growth was independent of autophagy. (A) Representative western blot images of autophagy markers (p62 and LC3 I/II) in U87 and U251 cell lines with VMP1‐OE. (B) Representative transmission electron microscopy images of a cell in U87 and U251 with VMP1‐OE, showing no differences in autophagosome formation. Top: scale bar, 2 µm. Bottom: scale bar, 500 nm. (C) Western blot images of tissue samples from our glioma cohort (glioma) (*n* = 47) and normal brain tissue (N), showing the protein expression of autophagy markers (p62, Beclin 1, and LC3 I/II). (D) Quantification of western blot images, patients were separated into two groups based on median VMP1 expression: VMP1^low^ glioma (*n* = 23) and VMP1^high^ glioma (*n* = 24). (E) Correlation analysis of western blot quantification value between VMP1 and autophagy markers (p62, Beclin 1, and LC3 I/II). (F) Confirmation of VMP1 knockdown in U87 and U251 cells using two different targeting sequences by western blot analysis. (G) U87 subcutaneous xenografts of VMP1 knockdown (shVMP1) and control vector (shNC) at Day 27 post‐injection (*n* = 10) (left), and the tumor volume measured from Day 14 to Day 27 (right). (H) Bioluminescence imaging of U87 shVMP1 and vector orthotopic xenografts at Day 28. (I) Representative images of hematoxylin and eosin‐stained sections at Day 28 post‐injection. Scale bar, 2000 µm. (J) Kaplan–Meier survival analysis of mice with U87 shVMP1 and vector intracranial xenografts (*n* = 5). (K) Representative western blot images of U87 shVMP1 and vector subcutaneous xenografts showing the expression of autophagy markers p62 and LC3 I/II (left). Quantification of band intensities normalized to GAPDH (right). ns, no statistical significance; ^*^ *p *< 0.05; ^**^ *p *< 0.01; ^***^ *p *< 0.001.

Next, to investigate the molecular mechanisms underlying the tumor‐promoting function of VMP1, we determined the effect of VMP1‐OE on autophagy. VMP1‐OE was not found to induce any effects on autophagy in GBM cells, as indicated by the unchanged expression of p62 and LC3 I/II (Figure 3A), nor did it cause any changes in the formation of autophagosomes, as no obvious autophagosomes were seen under electron microscopy (Figure 3B). Moreover, correlation analyses using our patient cohort revealed no dramatic difference in autophagy markers (p62, Beclin 1, and LC3 I/II) between VMP1^low^ and VMP1^high^ gliomas and no significant associations between VMP1 and the autophagy markers (Figure 3C–E), suggesting that the tumor‐promoting effects of VMP1‐OE were unlikely to be mediated by autophagy. We have also constructed VMP1 knockdown cell lines shVMP1‐1 and shVMP1‐2 targeting two different VMP1 sequences (Figure 3F). Consistently, VMP1 depletion in tumor cells (shVMP1) reduced tumor growth in vivo (Figure 3G–I and Figure S2) and prolonged animal survival when compared to control (shNC) (log‐rank *p *= 0.0002) (Figure 3J). Indeed, the functional change of VMP1 knockdown cells does not seem to be associated with autophagy as indicated by the unchanged level of p62 and LC3 I/II (Figure 3K), further validating that the oncogenic role of VMP1 is not likely to be mediated through autophagy.

### 2.3 VMP1 promotes Angiogenesis and Vascular Permeability via VEGFA‐VEGFR2 Signaling in Endothelial Cells

**FIGURE 4:**
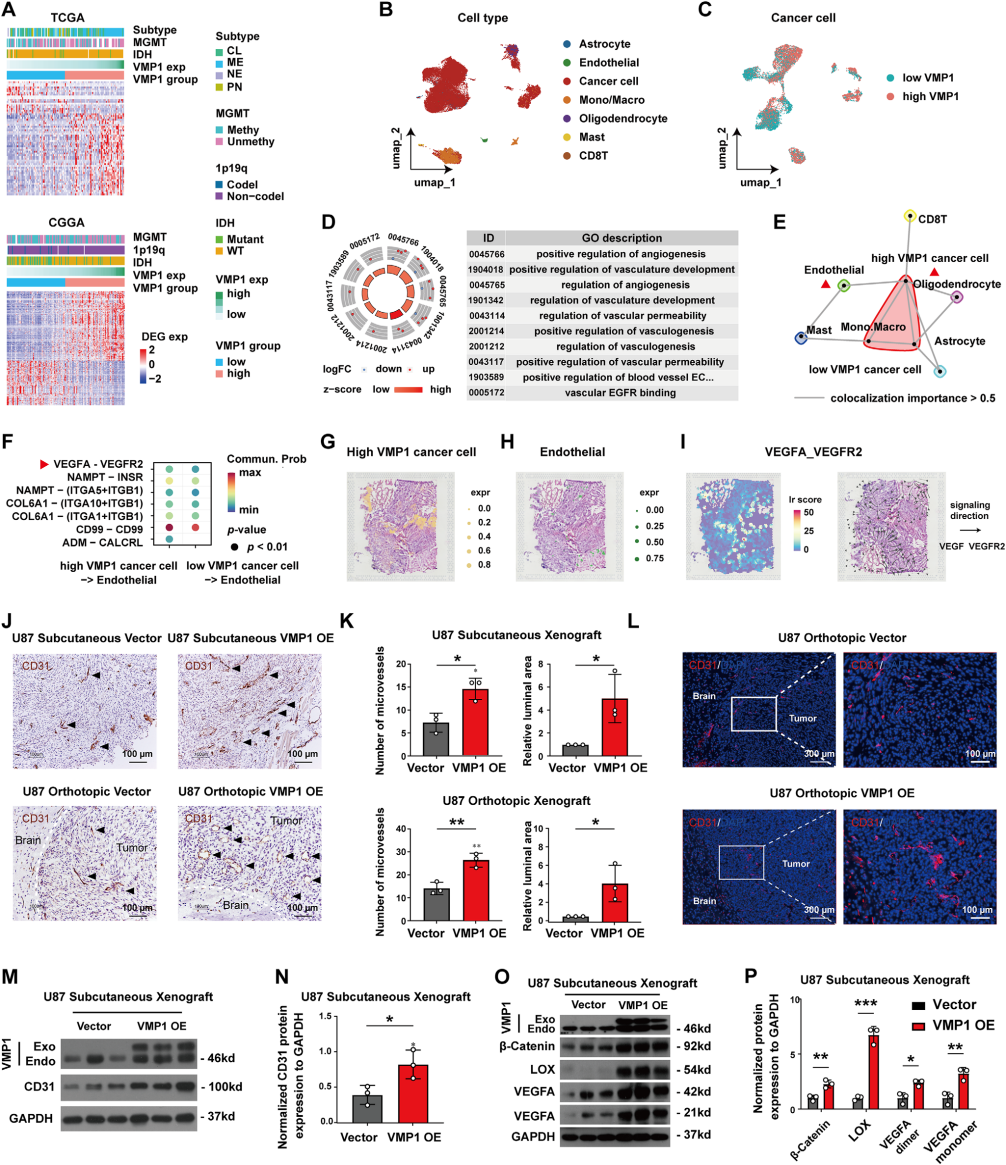
VMP1 promotes angiogenesis via VEGFA‐VEGFR2 signaling in endothelial cells. (A) Heatmap of differentially expressed genes between VMP1^low^ and VMP1^high^ glioblastoma with different subtypes, MGMT status, 1p19q status, and IDH status using TCGA and CGGA datasets. CL, classical; MES, mesenchymal; NE, neural; PN, pro‐neural. MGMT Methy, MGMT promoter methylated; MGMT Unmethy, MGMT promoter unmethylated; 1p19q Codel, co‐deletion; Non‐codel, non‐co‐deletion; IDH WT, wild type. (B) The UMAP plot of major cell populations in single‐cell mRNA datasets. Macro, Macrophage; Mono, Monocyte. (C) The UMAP plot of VMP1^low^ and VMP1^high^ cancer cells in single‐cell mRNA datasets. (D) GO enrichment of differentially expressed genes between bulk mRNA datasets (VMP1^low^ vs. VMP1^high^ glioblastoma tissues) and single‐cell mRNA datasets (VMP1^low^ vs. VMP1^high^ glioblastoma cells). (E) Network community plot for spatial co‐localization analysis of cell type abundance within one spot. The lines connecting cells represent strong colocalizations, with importance greater than 0.5. (F) Comparison of ligand‐receptor expression from VMP1^high/low^ cancer cells to endothelial cells in single‐cell datasets. (G, H) Spatial distribution of VMP1^high^ cancer cells and endothelial cells in spatial mRNA datasets. Expr, expression. (I) Spatial distribution of the VEGFA‐VEGFR2 signaling expression (left) and VEGFA‐VEGFR2 signaling direction (right). Lr, ligand and receptor. (J) Representative immunohistochemical (IHC) staining of endothelial marker CD31 in tissue sections. Black arrow indicates miceovessels with positive CD31 expression. The white dashed line represents the boundary between tumor and normal brain tissues. Scale bar, 100 µm. (K) Quantification of microvessel density (MVD) and the luminal area (LA) of microvessels in IHC staining of subcutaneous and orthotopic xenograft sections. (L) Representative immunofluorescence staining of CD31 expression (red) and DAPI (blue) in orthotopic xenografts. Scale bar, 300 µm (left). Scale bar, 100 µm (right). (M, N) Representative western blot image (M) and quantification (N) of CD31 protein expression in subcutaneous xenografts. (O, P) Representative western blot and quantification of angiogenesis‐promoting markers, including β‐catenin, lysyl oxidase (LOX), and VEGFA protein expression in subcutaneous xenograft. *p < 0.05; **p < 0.01; ***p < 0.001.

To identify the underlying non‐autophagic functions of VMP1 in promoting GBM cell proliferation, bioinformatic analyses using the bulk, single‐cell, and spatial transcriptomics data of patient GBM samples were performed. We identified 382 intersected differentially expressed genes (DEGs) (|log_2_(FC)| > 1, *p *< 0.05) between VMP1^low^ and VMP1^high^ GBM tissues in the TCGA and CGGA datasets (Figure 4A), and 707 DEGs (|log_2_(FC)| > 0.5, *p *< 0.05) between VMP1^low^ and VMP1^high^ GBM cells in the single‐cell datasets (Figure 4B,C). Comparison of the two gene sets revealed 33 overlapping genes, including *IGFBP3*, *CHI3L1*, and *SRGN*. Pathway analysis revealed that these common DEGs were predominantly enriched in angiogenesis‐related pathways (Figure 4D). Next, we asked whether VMP1 plays a role in the regulation of angiogenesis in GBM. By using co‐localization analysis, we showed that VMP1^high^ GBM cells were positioned in proximity to endothelial cells when compared to VMP1^low^ cells (Figure 4E and Figure S3). Cell‐cell communication analysis predicted that VMP1^high^ GBM cells are more likely to interact with endothelial cells relative to VMP1^low^ cells through VEGFA‐VEGFR2 signaling (Figure 4F). VEGFA has been recognized as an important angiogenesis‐promoting factor [27]. Consistent with our single‐cell analysis, spatial transcriptomics data also suggested the activation of VEGFA‐VEGFR2 signaling in VMP1^high^ GBM cells close to endothelial cells at the spatial level (Figure 4G–I).

To further investigate the role of VMP1 in GBM angiogenesis, in vivo experiments were conducted. The expression levels of endothelial marker CD31 on tissue sections obtained from subcutaneous and intracranial xenografts were assessed (Figure 4J). The extent of angiogenesis was semi‐quantified and presented as the microvessel density (MVD) and the luminal area (LA) of microvessels. We found that MVD and LA in U87 VMP1‐OE xenografts were significantly increased both in subcutaneous xenografts (MVD, *p *= 0.015; LA, *p = *0.029) and intracranial xenografts (MVD, *p =* 0.006; LA, *p = *0.036) (Figure 4K). Immunofluorescence staining of CD31 confirmed an increase in angiogenesis in VMP‐OE tumors, particularly at the peritumoral region (Figure 4L). Upregulation of CD31 expression was found in tumors grown within the brain environment as well as within the subcutaneous environment (Figure 4M,N). Furthermore, key angiogenesis‐promoting markers, β‐catenin, lysyl oxidase (LOX), and VEGFA [28] were markedly upregulated in VMP1‐OE tumors compared to their vector controls (Figure 4O,P).

**FIGURE 5:**
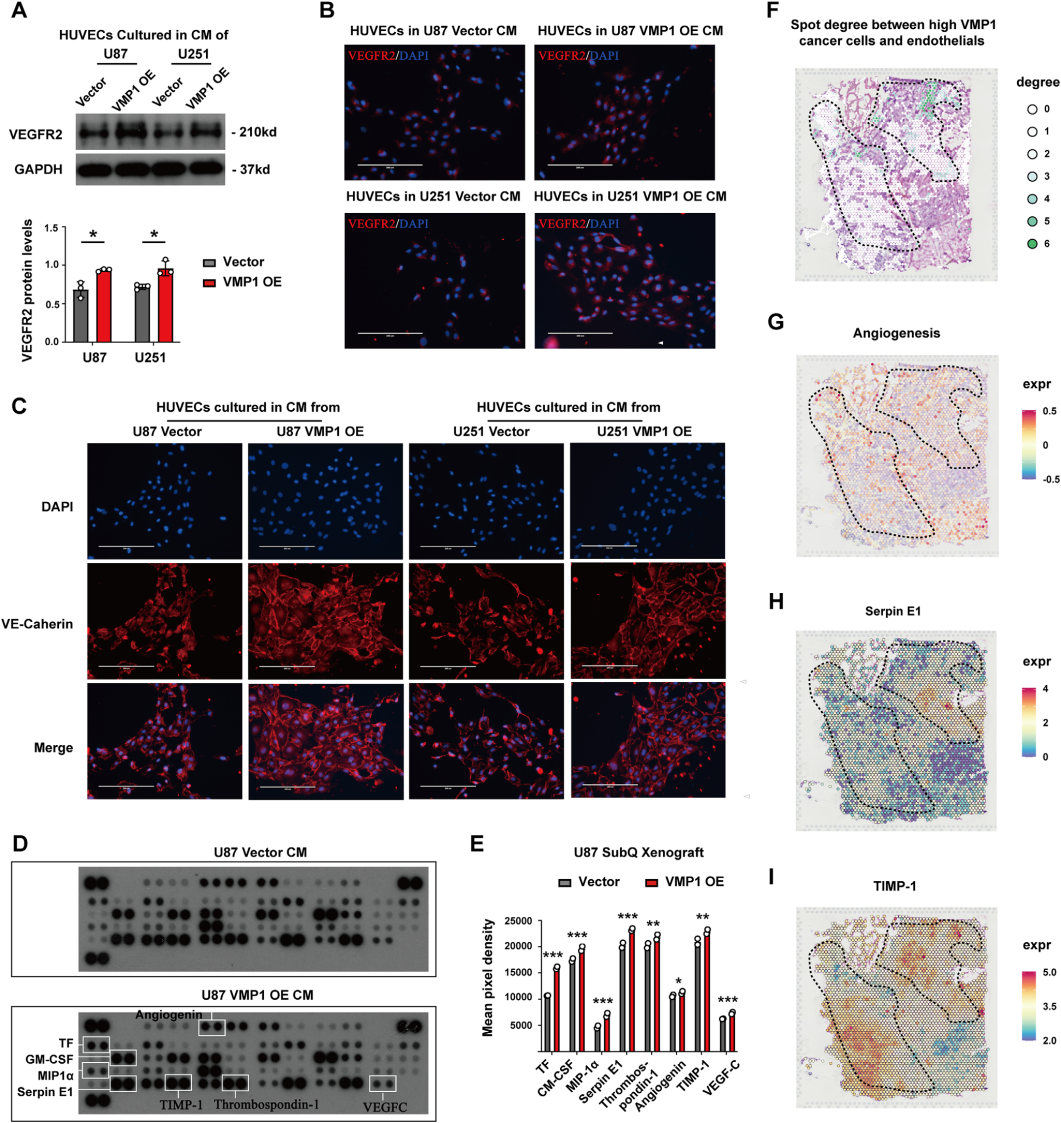
VMP1 mediates angiogenesis and vascular permeability through activation of endothelial cells in the TME. (A) Representative western blot image (top) and quantification (bottom) of VEGFR2 expression in human primary endothelial cells (HUVEC) after culturing with conditioned medium (CM) collected from VMP1‐overexpressing glioblastoma cell lines U87 and U251. (B) Representative immunofluorescence image of VEGFR2 expression (red) and DAPI (blue) in HUVEC cultured with conditioned medium. Scale bar, 200 µm. (C) Representative immunofluorescence staining image of VE‐cadherin expression (red) and DAPI (blue) in HUVEC with CM. Scale bar, 200 µm. (D) Human protein angiogenesis array showing 55 angiogenesis‐related proteins in the CM collected. (E) Quantification of eight of the angiogenesis‐related proteins, including tissue factor (TF), granulocyte‐macrophage colony stimulating factor (GM‐CSF), macrophage inflammatory protein 1α (MIP1α), Serpin E1, Thrombospondin‐1 (THBS1), Angiogenin, tissue inhibitor of metalloproteinase 1 (TIMP‐1), and VEGF‐C. (F) Spatial distribution of spot degree between VMP1^high^ cancer cells and endothelial cells in the spatial mRNA dataset. (G–I) Spatial distribution of angiogenesis (G), Serpin E1 (H), and TIMP1 (I) expression in the spatial mRNA dataset. *p < 0.05; **p < 0.01; ***p <0 .001.

We then examined whether and how VMP1 mediates interactions between tumor cells and endothelial cells using an indirect co‐culture system, where human primary endothelial cells (HUVEC) were incubated with conditioned medium (CM) collected from VMP1‐OE GBM cells. We found an upregulation of VEGFR2 in HUVECs co‐cultured with CM from VMP1‐OE cells (Figure 5A,B and Figure S4). In addition, these HUVECs demonstrated an irregular VE‐cadherin expression in endothelial cell‐cell contacts, indicating increased vascular permeability upon incubation with VMP1‐OE CMs (Figure 5C). These findings are strongly suggestive of the presence of soluble pro‐angiogenic factors secreted by VMP1‐OE GBM cells, which may then induce VEGFR2 expression in endothelial cells and attenuate endothelial junction stability. To identify the relevant secretory factors, CMs from control and VMP1‐OE cells were subjected to human protein angiogenesis arrays, which included 55 angiogenesis‐related protein markers. Eight out of 55 angiogenesis‐related proteins showed increased expression in the CM from VMP1‐OE cells (Figure 5D,E). These proteins included tissue factor (TF) (*p *< 0.0001), granulocyte‐macrophage colony stimulating factor (GM‐CSF) (*p = *0.0004), macrophage inflammatory protein 1α (MIP1α) (*p < *0.0001), Serpin E1 (*p < *0.0001), Thrombospondin‐1 (THBS1) (*p = *0.0049), Angiogenin (*p = *0.0170), tissue inhibitor of metalloproteinase 1 (TIMP‐1) (*p = *0.0032), and VEGF‐C (*p *< 0.0001). Among them, Serpin E1 and TIMP‐1 were observed to be highly co‐localized to the clustering regions between VMP1^high^ GBM cells and endothelial cells, which were highly angiogenic sites (Figure 5F–I). Collectively, our data suggest that VMP1^high^ GBM cells could enhance angiogenesis and vascular permeability by interacting with endothelial cells through the VEGFA‐VEGFR2 signaling pathway.

### 2.4 Targeted Inhibition of VEGFA Suppresses VMP1‐Mediated Tumor Growth

**FIGURE 6:**
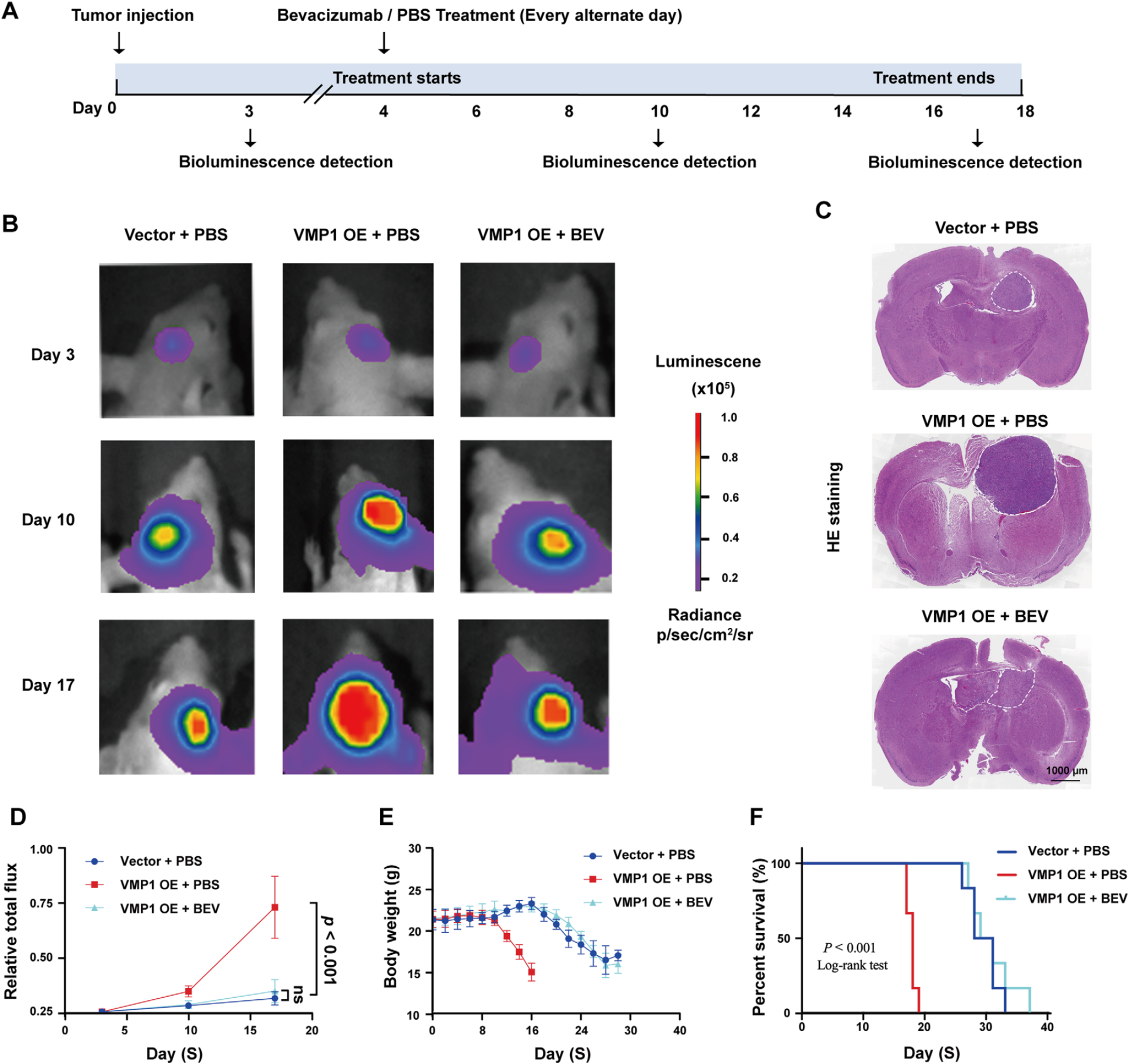
Targeted inhibition of VEGFA represses VMP1‐mediated tumor growth. (A) The treatment timeline and bioluminescence detection of mice treated with bevacizumab (BEV) at different time points (Day 3, Day 7, and Day 13). (B) Bioluminescence detection of U87 tumor‐bearing mice treated with BEV and vehicle. (C) Hematoxylin and eosin staining of mice brain, dotted area indicates the tumor region. Scale bar, 1000 µm. (D) Relative total photon flux in orthotopic mice model after treatment. (E) Changes in body weight in mice after treatments. (F) Kaplan–Meier survival of mice after being treated with bevacizumab and vector control. ns, no statistical significance.

Next, we asked whether VMP1‐mediated tumor growth was dependent on VEGFA. BEV, a monoclonal antibody that targets VEGFA to inhibit angiogenesis, was used to treat VMP1‐OE tumors in an orthotopic mice model (Figure 6A). After 2 weeks, VMP1‐OE + BEV treatment profoundly suppressed tumor growth compared to the vehicle control (Figure 6B–E) and prolonged the overall survival of mice (Figure 6F), suggesting that BEV could abrogate the VEGFA‐dependent oncogenic effect in VMP1‐driven tumors.

## 3 Discussion

Macroautophagy (“autophagy,” hereafter) is the primary form of autophagy involving the formation of an autophagosome, which is critical for maintaining cellular homeostasis and protection against various forms of cellular damage [29]. Cancer cells may also exploit the autophagic mechanism through different pathways to support cell survival [12]. For example, autophagy can provide an internal source of nutrients and energy during periods of nutrient deprivation; it promotes the degradation of unfolded proteins, which may in turn contribute to drug resistance; it also provides a means for metabolic reprogramming to support rapid cell proliferation [14, 15, 30]. Yet, cancer cells may exhibit varying levels of autophagy depending on a host of intricate factors such as tumor staging, tumor type, and TME [16]. While many cancer cells indeed demonstrate high levels of autophagic activity, there are instances where cancer cells would exhibit dysregulated or diminished autophagy, particularly in those that harbor genetic aberrations in autophagy genes [14]. As such, the role of autophagy in cancer is widely acknowledged to be context‐dependent and may range from being tumor‐promoting to tumor‐suppressing, as is the case for VMP1 across various cancer types.

Driven by our initial observation of poor clinical outcomes in patients with high VMP1‐expressing GBMs, we sought to understand the role of VMP1 in glioma progression. Our findings partly align with previous study by Lin et al. on the tumor‐promoting role of VMP1, which demonstrated growth impairment in VMP1‐depleted GBM cells through blockade of autophagic flux [31]. However, we did not find evidence of altered autophagic activities following forced VMP1‐OE, and surmised that VMP1‐driven tumor progression is, at least partially, independent of autophagic function without additional autophagy activation trigger. Indeed, the TMEM protein family, to which VMP1 belongs, fulfills important physiological functions encompassing autophagy, immune response, cellular differentiation, and more [2]. Growing evidence indicates that a large number of TMEM proteins are implicated in cancer development and may serve as potential prognostic markers [32]. Certain TMEM proteins are involved in promoting endothelial cell survival and the regulation of endothelial cell migration by interacting with the linker of nucleoskeleton and cytoskeleton complex (LINC complex) [33, 34]. A recent study also described a previously unreported role of VMP1 in regulating the release of proinflammatory molecules in neurodegenerative diseases, in support of a non‐autophagic function of VMP1 in cancer pathogenesis [35].

The present study thus provides the first piece of evidence that VMP1 can promote angiogenesis through VEGFA‐VEGFR2 signaling and endothelial cell activation, and that VMP1‐OE GBM cells could activate the angiogenic switch by upregulating VEGFR2 expression, possibly through the production of soluble pro‐angiogenic factors. VEGFA plays a dominant role in VEGF signaling‐mediated angiogenesis. Upon binding to VEGF receptors on endothelial cells, VEGFA initiates strong mitogenic and survival signals exclusive to VEGFR2 [36]. In response, activated endothelial cells would proliferate and move towards the signal source, thereby extending the blood vessels. We also found that the enhanced neovascularization was accompanied by an increase in vascular permeability at endothelial cell‐cell junctions, both of which are hallmarks of disease progression and poor prognosis in GBM patients [37, 38, 39, 40]. It is therefore likely that the TME, and in particular the tumor vasculature, represents a context‐dependent factor essential for the functional interpretation of VMP1 in cancer.

Our findings were further reinforced by using therapeutics that target VEGFA in a VMP1‐driven tumor model. BEV is an FDA‐approved anti‐VEGFA antibody that has been used as a second‐line treatment for recurrent GBM. While BEV has been shown to provide benefit in improving quality of life and shrink tumors in some cases, its impact on overall survival is limited [41, 42, 43]. In this study, we showed that the use of BEV can abrogate the tumor‐promoting effect of VMP1 in GBM, and that targeting VMP1 or VMP1‐associated pathways may serve as a novel therapeutic approach, either in combination with or as an alternative to BEV. As we begin to understand more about the intricate interplay between VMP1, autophagy, and tumor progression, the development of more personalized treatments that specifically target the essential survival mechanisms may bring new promises. Our finding that VMP1‐driven tumor growth may be autophagy‐independent in GBM is consistent with the prevailing notion that in most cancers, autophagy would predominantly function as a survival mechanism rather than a primary driving force of cancer growth [44] and that a context‐specific approach should be adopted in future studies and in other cancer types.

A limitation of the present study is that primary GBM or patient‐derived xenograft models, which better recapitulate the brain TME, were not included, and whether VMP1‐mediated angiogenesis occurs in other cancer types remains to be explored. Nevertheless, our findings reveal that high VMP1 expression predicts poor survival in GBM and drives pro‐angiogenic tumor growth independently of autophagy via VEGFA‐VEGFR2 signaling. Importantly, this VMP‐1 mediated angiogenesis can be effectively inhibited by BEV, suggesting that VMP1 may serve as a biomarker to stratify patients for individualized anti‐angiogenic therapy.

## 4 Materials and Methods

### 4.1 Human Glioma Specimens and Ethics Statement

Forty‐seven glioma tissues and four non‐tumor brain tissues were collected at Queen Mary Hospital, The University of Hong Kong, between 2006 and 2016. Specimens were snap‐frozen in liquid nitrogen after resection and were fixed immediately in 10% neutral buffered formalin. All glioma specimens had a confirmed diagnosis by two independent specialists in pathology. Written informed consent for research purposes was obtained from all subjects or legal guardians. The study protocol was reviewed and approved by our institution review board (HKU/HA HKW IRB, approval no. UW 07–273).

### 4.2 Cell Lines and Culture Conditions

Human embryonic kidney cells 293T (293T) and human GBM cell lines U87 and U251 were purchased from the American Type Culture Collection (ATCC). Both GBM cell lines were authenticated by short‐tandem repeat [45] profiling (Genetica). Primary human umbilical vein endothelial cells (HUVECs) from a pool of donors were purchased from Lonza. 293T cells were cultured in Dulbecco's Modified Eagle's Medium (DMEM) while U87 and U251 cells were cultured in Minimum Essential Medium (MEMα) supplemented with 10% heat‐inactivated fetal bovine serum (FBS) (Gibco) and 1% penicillin/streptomycin. HUVECs (Passages 2–4) were cultured in EBM‐2 basal medium (Lonza) supplemented with 2% FBS and growth factors included in the EGM‐2 BulletKit (Lonza). Cell cultures were maintained at 37°C in a humidified incubator under air/CO_2_ (95:5, v/v) atmosphere.

### 4.3 Public Data Acquisition of Glioma Samples

The bulk mRNA expression data were downloaded from publicly available databases through their official portals, including The Cancer Genome Atlas (TCGA) glioma and GBM cohorts accessed via the UCSC Xena platform, the Chinese Glioma Genome Atlas (CGGA), and the Gene Expression Omnibus (GEO; GSE16011). Only open‐access, de‐identified datasets that do not require controlled authorization were used. All bulk transcriptomics data were accessed on December 17, 2021. Single‐cell mRNA expression data were obtained from the GEO database, including GSE138794, GSE139448, and GSE141982 (accessed on June 19, 2024). Spatial mRNA expression data were obtained from the 10× Genomics official platform (Human Glioblastoma: Whole Transcriptome Analysis, Spatial Gene Expression Dataset by Space Ranger 1.2.0; accessed on June 19, 2024). According to the corresponding database documentation, all datasets consist of de‐identified data and are not subject to controlled access or known export control restrictions. Data were filtered, normalized, and integrated using the “Seurat” and “Harmony” packages [46], and cell types were annotated based on previously published studies [47, 48, 49]. All analyses were conducted in compliance with applicable data‐sharing and international data transfer regulations, and no raw data were redistributed.

### 4.4 Plasmids and Establishment of Stable Cell Lines

The mammalian lentiviral VMP1‐overexpressing clone (pRRL‐SFFV‐VMP1‐Myc‐Flag‐iGFP) and its control vector (pRRL‐SFFV‐iGFP) were kind gifts from Prof. Edo Vellenga (Department of Hematology, Cancer Research Centre Groningen, University Medical Centre Groningen, University of Groningen) [50]. For stable knockdown of VMP1, luciferase‐expressing lentiviral vectors containing short‐hairpin RNA (shRNA) targeting human VMP1 sequence were used and were obtained from GenePharma. Plasmid transfection and lentivirus production were performed using PolyJet (SignaGen) transfection reagent. For real‐time monitoring of intracranial tumor growth, luciferase‐expressing U87 cells (pGLV6/Firefly/Puro; GenePharma) were used as parental cells.

### 4.5 Protein Extraction and Western Blot Analysis

Protein extraction and western blot analysis were performed as previously described [51]. A total of 20 µg of total protein lysate was subjected to 10% SDS‐PAGE gel electrophoresis and transferred to PVDF membranes (Bio‐Rad Laboratories). Membranes were blocked with 5% non‐fat milk for 1 h at room temperature and then incubated with primary antibodies at 4°C overnight. Primary antibodies used are listed in Table S1. Secondary antibodies (1:5000) were used to incubate the membranes at room temperature for 1 h. After washing with TBST, immunoblots were developed by enhanced chemiluminescence (ECL) detection system with x‐ray film visualization. Band densities were quantified by ImageJ software. Relative protein expressions were determined by normalizing the densitometry values of the protein of interest to those of GAPDH.

### 4.6 Preparation of CM

Vector or VMP1‐overexpressing GBM cells (U87 and U251) were routinely cultured in T75 culture flasks. At cell confluency of 80%, culture medium was discarded, and the cells were washed with PBS twice. A total of 10 mL of FBS‐free medium was then added for subsequent incubation. CM was collected after 48 h and filtered through a 0.2 µm pore size filter (PALL Corporation). CM was aliquoted and stored at −80°C until use.

### 4.7 Immunofluorescence Staining

Immunofluorescence staining was performed on cell cultures or cryosections as previously described [28, 51]. Briefly, HUVECs were washed with PBS and fixed with 4% PFA after incubation with CMs for 48 h. Cryosections were hydrated in TBST, followed by permeabilization with 0.5% Triton‐X. Then, the cells or cryosections were blocked with 3% BSA for 30 min at room temperature, followed by incubation with primary antibodies (VEGFR2, 1:200; CD31, 1:50; VE‐cadherin, 1:200) at 4°C overnight. On the next day, cells or cryosections were incubated with fluorescein‐conjugated secondary antibodies at a dilution of 1:500 for 1 h at room temperature. Immunofluorescence images were acquired under a fluorescent microscope (EVOS, Life Technologies).

### 4.8 Immunohistochemical Staining

Immunohistochemical (IHC) staining was performed on paraffin‐embedded sections and cryosections. VMP1, CD31, and Ki67 antibodies were diluted at 1:50, 1:50, and 1:100, respectively. After overnight incubation with primary antibody, tissue sections were incubated with horseradish peroxidase (HRP)‐conjugated secondary antibodies (DAKO) for 30 min at room temperature. Signals were then detected with DAKO EnVison+ system using the 3,3’‐diaminobenzidine (DAB) chromogen substrate, followed by counterstaining with hematoxylin. Images were taken under a light microscope (Nikon). The histochemistry score was applied to assess VMP1 expression in clinical glioma specimens, and GBM patients were divided into VMP1^high^ expression score and VMP1^low^ expression score groups using the median IHC score of VMP1 as the cut‐off value. Proliferation index was determined by dividing the number of Ki67‐positive cells by the total number of hematoxylin‐positive cells in at least three random fields at 200× magnification. MVD was evaluated in CD31‐stained sections. CD31‐positive endothelial cells or endothelial cell clusters clearly separated from adjacent microvessels were counted as a single microvessel. The number of microvessels in at least 5– 10 random 100× microscopic fields in tumor sections from three or more xenograft tumors was used to compare the difference in MVD between two groups. LA of microvessel was also evaluated and calculated using the ImageJ software.

### 4.9 Protein Angiogenesis Array

The expression levels of angiogenesis‐related factors in the conditioned media were detected with the human angiogenesis array kit (R&D Systems). The nitrocellulose membrane contains 55 different capture antibodies printed in duplicate. Briefly, the membranes were blocked for 1 h in a 4‐well multi‐dish at room temperature, followed by incubation with mixture of CM and reconstituted detection antibody cocktail overnight at 4°C. After three washes, the membranes were incubated with diluted streptavidin‐HRP for 30 min at room temperature. Signal was developed by ECL detection system with x‐ray film visualization. Spot densities were quantified by ImageJ software. Differentially expressed protein markers were further verified by western blot with lysates obtained from subcutaneous xenografts.

### 4.10 Differential Gene Expression and Gene Set Enrichment Analyses

The 50% median value of VMP1 mRNA expression was set as the cutoff to divide GBM bulk or GBM cancer‐cell samples into VMP1^low^ and VMP1^high^ groups. “Limma” package and “Seurat” package were separately used for identifying the DEGs. Afterwards, the intersection of DEGs between bulk and cancer‐cell samples was screened and enriched for the Gene Ontology (GO) analysis. Furthermore, apoptosis signatures were retrieved from MsigDB v7.0 (M14493; https://www.gsea‐msigdb.org/gsea/msigdb). The spatial distribution of angiogenesis was quantified in the spatial transcriptome using “GSVA” package [52].

### 4.11 Spatial Co‐Localization Analysis

“FindTransferAnchors” function of “Seurat” package was used to map each cell type to the spatial region.

Spatial co‐localization of these cells was computed by “mistyR” package [53], from the intrinsic (intra) view that measures the abundance of cell type within one spot [54]. In addition, homotypic scores (spot degree) were also calculated to assess the extent of spatial clustering between VMP1^high^ cancer cells and endothelial cells according to a previously proposed method [55].

### 4.12 Intercellular Interaction Analysis

“Cellchat” [56] and “stlearn” [57] packages were separately used to analyze cell‐cell interaction in both single‐cell and spatial mRNA expression data. The expression of ligand‐receptor pairs was compared between VMP1^low^ and VMP1^high^ cancer cells. The direction of interaction was also visualized in the spatial data using “Commot” package [58].

### 4.13 Transmission Electron Microscopy

U87/U251 Vector and VMP1‐OE cells were fixed with 2.5% glutaraldehyde in 0.1 M cacodylate buffer for 2 h. After washing, cells were treated with 1% osmium tetroxide and 0.1 M cacodylate. The fixed cells were dehydrated following various concentrations of ethanol and embedded in epoxy resin. Cellular structures were observed, and images were obtained under the Hitachi TEM system at 80 kV.

### 4.14 Subcutaneous and Orthotopic Xenograft Models

Male athymic BALB/c nude mice (aged 5–6 weeks) were obtained from the Centre for Comparative Medicine Research (CCMR) of The University of Hong Kong and Guangdong Province. All operations and animal care were performed in accordance with the guidelines approved by the Committee on the Use of Live Animals for Teaching and Research (CULATR) of The University of Hong Kong (5403‐20) and Guangdong Provincial People's Hospital (Guangdong Academy of Medical Sciences), Southern Medical University (S2022‐238‐01). Mice were kept in a 12 h light‐dark cycle with enrichment and ad libitum access to water and chow. The animal holding room was kept at 19 ± 2°C, with humidity maintained at 44 ± 2%. For subcutaneous tumor model, U87 cell suspension (1 × 10^6^) in 100 µL serum‐free medium was injected into the right flanks of nude mice. Tumor volume was calculated according to the formula: *V* = ½ *ab* ^2^ with *a* and *b* representing the longest and shortest diameter, respectively. Measurements were recorded weekly. Tumor weight was also measured after isolation. For intracranial tumor model, mice were first anesthetized by intraperitoneal injection of ketamine (80 mg/kg) and xylazine (10 mg/kg) and fitted onto the stereotaxic device. A small skin incision was made to expose the bregma suture, and a burr hole was created at 1 mm anterior and 2 mm lateral to the bregma with a micromotor drill. U87 cells (5 × 10^5^) resuspended in 2 µL serum‐free medium were transferred to a 26‐gauge Hamilton syringe (Hamilton) and slowly (0.4 µL/min) injected into the right striatum at a depth of 2.5 mm after 0.5 mm withdrawal.

Tumor size was semi‐quantitatively represented by the bioluminescence signal value detected by the PE IVIS Spectrum in vivo imaging system (PerkinElmer), with the reporter substrate D‐Luciferin (Gold Biotechnology) administered intraperitoneally at 100 mg/kg. BEV was administered at 10 mg/kg iv every other day from Day 4 post‐implantation. Mice were sacrificed at the onset of cachexia or when the largest diameter of the tumor reached 17 mm. Survival time was also recorded and subject to Kaplan–Meier survival analysis.

### 4.15 Statistical Analysis

Data were expressed as mean ± SD. Comparisons between two groups were performed by Student's *t*‐test, while differences among three or more groups were assessed by one‐way analysis of variance (ANOVA) followed by Tukey's multiple comparison test. Associations between two variables were computed using the Pearson correlation coefficient. Survival analysis was performed using the Kaplan–Meier method, followed by the log‐rank test (Mantel–Cox). Statistical analyses were performed using GraphPad Prism 7 (GraphPad Software, San Diego, CA), SPSS version 13.0 (SPSS Inc, Chicago, IL), R 4.3.1 (https://www.r‐project.org/about.html), and Python 3.9 (https://www.python.org). Tests were 2‐tailed, and *p *< 0.05 was considered statistically significant.

## Author Contributions

L.J. and G.L. conceptualized the study. L.J., B.C., W.G., Z.Z., and K.K. developed the methodology. L.J., B.C., and J.L. performed the formal analysis and data visualization. L.J., B.C., and K.K. performed the investigation. L.J., B.C., and K.K. wrote the original draft. W.G., S.A.,P.W., and K.K. wrote, reviewed, and edited the manuscript. Z.Z., G.L., and K.K. provided the resources. G.L., and K.K. supervised the study. K.K. and P.W. secured funding. All authors have read and approved the final manuscript.

## Funding

This work was supported by research funding from the National Natural Science Foundation of China (Grant Nos. 81201995 and 82403157) and Guangzhou Science and Technology Plan Project (Grant No. 2023A04J0525).

## Ethics Statement

The study was approved by the Institutional Review Board of The University of Hong Kong/Hospital Authority Hong Kong West Cluster (HKU/HA HKW IRB) (approval no. UW 07–273). Informed consent was obtained from all participants in the study. Animal works were performed in accordance with the guidelines approved by the Committee on the Use of Live Animal for Teaching and Research (CULATR) of the University of Hong Kong (5403‐20) and Guangdong Provincial People's Hospital (Guangdong Academy of Medical Sciences), Southern Medical University (S2022‐238‐01).

## Conflicts of Interest

The authors declare no conflicts of interest.

## Supporting information




**Supporting Figure 1**: VMP1 expression profiling in IDH‐wildtype glioblastoma. Quantitative stratification of 19 cases by VMP1 IHC intensity: low‐expression group (n = 9) and high‐expression group (n = 10). ^***^ *p*<0.001.
**Supporting Figure 2**: Relative total photon flux of bioluminescence in mice with U87 shVMP1 and vector at day 28 post‐injection. ^*^ *p*<0.05.
**Supporting Figure 3**: Heatmap showing the cell‐cell colocalization relationship within a spot. Endothelial cells (red boxes) show stronger colocalization with VMP1^high^ cancer cells than with VMP1^low^ cancer cells.
**Supporting Figure 4**: Quantification of VEGFR2 red‐fluorescence intensity (IntDen) per HUVEC cultured with conditioned medium from VMP1‐overexpressing glioblastoma cell lines U87 and U251.
**Supporting Table 1**: Sources of antibodies used in this study.

## Data Availability

All datasets generated in this study are available upon reasonable requests to the corresponding author.
