# Supplementary material for: Integrative Single‐Cell Analysis Reveals Targetable Vacuole Membrane Protein 1‐Mediated Mechanism of Tumor Angiogenesis in Glioblastoma
Source: MedComm (2020). 2026 Jan 25;7(2):e70619. doi: 10.1002/mco2.70619 (PMC12832072; doi:10.1002/mco2.70619)
Supplement: Supplementary file 1 — Supporting Figure 1: VMP1 expression profiling in IDH‐wildtype glioblastoma. Quantitative stratification of 19 cases by VMP1 IHC intensity: low‐expression group (n = 9) and high‐expression group (n = 10). *** p<0.001. Supporting Figure 2: Relative total photon flux of bioluminescence in mice with U87 shVMP1 and vector at day 28 post‐injection. * p<0.05. Supporting Figure 3: Heatmap showing the cell‐cell colocalization relationship within a spot. Endothelial cells (red boxes) show stronger colocalization with VMP1high cancer cells than with VMP1low cancer cells. Supporting Figure 4: Quantification of VEGFR2 red‐fluorescence intensity (IntDen) per HUVEC cultured with conditioned medium from VMP1‐overexpressing glioblastoma cell lines U87 and U251. Supporting Table 1: Sources of antibodies used in this study. [file MCO2-7-e70619-s001.pdf]

Integrative single-cell analysis reveals targetable vacuole membrane protein 1-mediated mechanism of tumor angiogenesis in glioblastoma

*Lei Jin[1,5,#], Bo Chen[5,#], Junbo Liao[5], Wenlong Guo[1], Zhiyuan Zhu[2,5], Salida Ali[5], Gilberto Ka-Kit Leung[3,4,5], Peng Wang[1,**], Karrie M. Kiang[5,6,*]*

[1]Department of Neurosurgery, Guangdong Provincial People's Hospital, Guangdong Academy of Medical Sciences, Southern Medical University, Guangzhou, China

[2]Department of Functional Neurosurgery, Zhujiang Hospital, Southern Medical University, Guangzhou, China

[3]Queen Mary Hospital, Hospital Authority, Hong Kong SAR, China

[4]Clinical Neuroscience Consortium, LKS Faculty of Medicine, The University of Hong Kong, Hong Kong SAR, China

[5]Department of Surgery, School of Clinical Medicine, LKS Faculty of Medicine, University of Hong Kong, Hong Kong SAR, China

[6]Lead contact

[#]These authors contributed equally.

[*]Correspondence: Dr. Karrie M. Kiang, Department of Surgery, School of Clinical Medicine, LKS Faculty of Medicine, University of Hong Kong, 21 Sassoon Road, Pokfulam, Hong Kong, China. Email: mykiang@hku.hk, Phone: (852) 3917 9649, Fax: (852) 3917 9634, ORCID: 0000-0002-4354-9648

[**]Co-correspondence: Dr. Peng Wang, Department of Neurosurgery, Guangdong Provincial People's Hospital, Guangdong Academy of Medical Sciences, Southern Medical University, 106 Zhongshan 2nd Road, 510080, Guangzhou, China. Email: wangpeng_82@sina.com, Phone: 86 (020) 8382 7812.

Running Head: VMP1 mediates angiogenesis in glioblastoma

Supplementary Figures

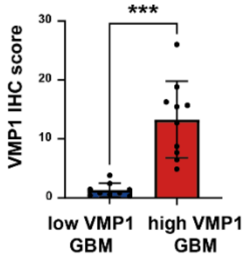

Figure S1. VMP1 expression profiling in IDH-wildtype glioblastoma. Quantitative stratification of 19 cases by VMP1 IHC intensity: low-expression group (n=9) and high-expression group (n=10). $^{***}p<0.001$.

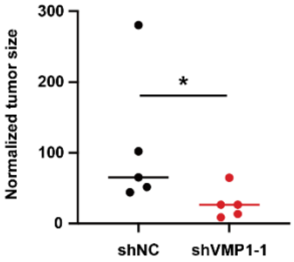

Figure S2. Relative total photon flux of bioluminescence in mice with U87 shVMP1 and vector at day 28 post-injection. $^{*}p<0.05$.

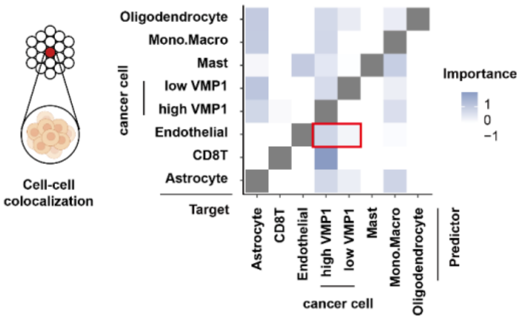

Figure S3. Heatmap showing the cell-cell colocalization relationship within a spot. Endothelial cells (red boxes) show stronger colocalization with VMP1^{high} cancer cells than with VMP1^{low} cancer cells.

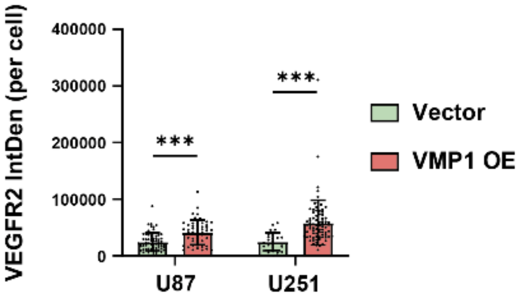

Figure S4. Quantification of VEGFR2 red-fluorescence intensity (IntDen) per HUVEC cultured with conditioned medium from VMP1-overexpressing glioblastoma cell lines U87 and U251.

Supplementary Table

Supplementary Table S1. Sources of antibodies used in this study.

Antibodies	Source	Cat No.
anti-VMP1	Cell Signaling Technology	12929S
anti-Beclin 1	Cell Signaling Technology	3495S
anti-P62	Cell Signaling Technology	5114S
anti-LC3	Cell Signaling Technology	4108S
anti-GAPDH	Cell Signaling Technology	5174S
anti-β-Catenin	Cell Signaling Technology	8480T
anti-LOX	Cell Signaling Technology	58135S
anti-Ki67	Cell Signaling Technology	9449S
anti-VEGFR2	Cell Signaling Technology	9698S
anti-STAT3	Cell Signaling Technology	12640S
anti-p-STAT3	Cell Signaling Technology	9134S
anti-VEGFA	Santa Cruz Biotechnology	sc-152
anti-VE-Cadherin	Thermo Fisher Scientific	36-1900
anti-CD31	Abcam	Ab28634
